# Beyond challenges and enrichment: a qualitative account of cross-cultural experiences of nursing patients with an ethnic minority background in Norway

**DOI:** 10.1186/s12912-022-01102-x

**Published:** 2022-11-23

**Authors:** Tariq Alkhaled, Gudrun Rohde, Birgit Lie, Berit Johannessen

**Affiliations:** 1 Department of Health and Nursing, Faculty of Health and Sport Sciences, University of Agder, Kristiansand, Norway; 2 Department of Clinical Research, Sorlandet Hospital, Kristiansand, Norway

**Keywords:** Cross-cultural nursing, Communicational barriers, Cultural challenges, Ethnic minority, Cultural competence, Cultural sensitivity

## Abstract

**Background:**

Substantial mass migrations to the Scandinavian countries have made them heterogeneous and multicultural societies. Migration has also influenced the workforce, especially the healthcare system that has had to accommodate a culturally diverse population. This qualitative study aimed to explore nurses’ experiences in caring for patients with an ethnic minority background.

**Methods:**

Focus group interviews with a total of 21 nurses were conducted. Thematic analysis was performed on the transcribed and translated interviews.

**Results:**

The findings of this study revealed three major themes: various experiences concerning language barriers and the use of interpreters, the impact of religious and cultural values, and caring for patients with an ethnic minority background is professionally interesting but demanding.

**Conclusions:**

Caring for culturally diverse patients offers both challenges and opportunities to enhance cultural competence and cultural sensitivity among nurses. Various culturally divergent needs of patients such as family visits, food preferences, expression of pain, and gender sensitivity are interlinked and depend on effective communication during encounters with nurses and the healthcare system at large.

## Background

In recent decades, global migration has gradually grown. In 2021, more than 275 million individuals lived in a nation other than their native country [1]. The new population structure requires nurses to be familiar with intercultural, communicational, and institutional transactions between immigrants and the healthcare system [2, 3]. Hence, nursing now intersects readily with different cultural and social backgrounds [4–7]. Recognizing cultural diversity is critical for providing effective, safe, and compassionate care for patients with an immigrant background [8, 9]. Furthermore, understanding patients’ beliefs, values, and cultural attitudes is an important aspect of providing holistic care [9–11].

Previous studies show that nurses’ experiences in providing care for patients with an ethnic minority background consumes more time and energy than native patients [12–14]. Most of these challenges are cultural and communicational ones [10, 15–17]. Patient care may be negatively impacted by the presence of cultural and communication barriers between patients and nursing staff [18–20]. Miscommunication often causes unnecessary mistakes, inadequate care, and even death [21, 22]. For the vast majority of nurses, poor communication is the most important barrier to effective and comprehensive care [23–26]. This may lead to negative consequences including moral pressures, alienation, and disruption of workplace morale, while effective communication has a series of advantages including greater satisfaction, positive evaluation, treatment compliance, and good health outcomes [16, 27, 28]. Cultural competence and cultural sensitivity in nursing are linked to successful communication with patients [29, 30].

In the experience of Purnell and Fenkl (2019), health is both a socially constructed and a culturally relative concept [31]. Often cultural barriers revolve around acceptable ways of expressing pain, accepting physical support from the nursing staff, and desiring family members as visitors [32–34]. Similarly, food and gender-based biases of patients are cross-culturally determined factors.

### The Norwegian context

Many Norwegian cities are host to a significant proportion of non-Western immigrants, especially since the 1990s. In Norway, immigrants account for 18.4% of the total population [35]. All citizens of Norway have equal access to the public healthcare system, which is supported by taxes [36, 37]. According to the National Strategy on Immigrants’ Health, immigrants who do not speak Norwegian have the right to a free interpreter service during medical consultations [38].

There are limited studies on nurses’ cross-cultural experiences in the context of Norwegian hospitals. This deficit reflects the problems and difficulties of catering to the needs of immigrant patients. In summarizing the findings of previous studies, Høye and Severinsson (2010) found that professional nursing care faces the challenges of coping with immigrant patients’ culturally based communication problems and that cultural norms of patients are often in conflict with the clinical environment of “total care” [39]. Linguistic, cultural, and ethnic interactions were linked with job related stressors in the intensive care units of Norwegian hospitals [40]. On the other hand, Debesay et al. (2014) observed that nurses found it particularly stressful to deal with issues of intimate care and religiosity in the context of community nursing [41].

Hence, the aim of the present study was to explore nurses’ experiences in caring for patients with an ethnic minority background.

## Methods

### Study design

A qualitative exploratory design with focus group interviews was adopted to explore nurses’ experiences in caring for patients with an ethnic minority background. A focus group interview promotes a group process that can assist participants to clarify and explain their experiences in ways in which individual interviews might not be able to [42]. More specifically, the setting of focus group design allows researchers to avoid a “lone ranger” approach and achieve a more reflexive insight into nurses’ experiences. This kind of group setting encourages elaboration and probing because speaking in a group context confers empowerment [43]. This format was selected over individual interviews because discussing difficult or controversial subjects with colleagues present is typically easier on participants. Rich data were expected because the individuals were familiar with one another from working together. Individuals who are known to each other often add to the comments of others or provide opposing opinions, which can broaden and deepen the discussion [43].

### Recruitment process

The head nurse at a hospital in Norway was contacted and informed about the study. She distributed the written detailed information and informed consent forms in Norwegian among nurses. Reaching the target population was challenging, so we also used a snowball sampling strategy to recruit some of the participants [44, 45]. Nurses who showed interest in participating were asked for their informed consent and their telephone number or E-mail address. They were then contacted by the researchers to decide an appropriate place and time for the interviews to take place. They each received a small gift card as compensation for their time.

### Participants

Purposive sampling was used. A total of 21 registered nurses were recruited from a hospital in Norway. The inclusion criteria were as follows: (1) registered nurses who had worked in the hospital for a minimum of two years and had experiences with patients with an ethnic minority background, and (2) a willingness to share their experiences during clinical nursing care. The exclusion criterion consisted of nurses who had less than two years’ experience. Four of the participants had an ethnic minority background. Four had worked in other countries and eight had been in a student exchange program. The participants had 2- to 31-year experience as a nurse, fifteen of them had a bachelor’s degree, and six had a master’s degree. Table 1 shows the basic demographic data of the study participants.

**Table 1:** Characteristics of the participants

Group (No.)	Participant	Gender	Educational level	Years of experience	Country of origin	Worked in another country	Student exchange program	Number of languages spoken
Group 1 (4)	1	F	Bachelor	22	Denmark	Yes	-	3
	2	F	Master	12	Norway	-	Yes	3
	3	F	Bachelor	18	Norway	-	-	2
	4	F	Bachelor	32	Germany	Yes	-	4
Group 2^a^ (1)	5	F	Bachelor	14	Norway	Yes	-	5
Group 3 (4)	6	E	Master	11	Norway	-	Yes	2
	7	M	Bachelor	3	Norway	-	-	2
	8	F	Bachelor	16	Norway	-	Yes	2
	9	F	Bachelor	19	Norway	-	Yes	3
Group 4 (3)	10	F	Mater	21	Norway	-	Yes	2
	11	F	Master	30	Norway	-	Yes	2
	12	F	Bachelor	31	Norway	Yes	-	3
Group 5 (4)	13	F	Bachelor	3	Norway	-	Yes	2
	14	F	Bachelor	2	Norway	-	-	2
	15	F	Bachelor	3	Norway	-	-	2
	16	F	Bachelor	10	Cambodia	-	-	3
Group 6 (5)	17	M	Bachelor	3	Norway	-	-	2
	18	F	Master	16	Norway	-	Yes	3
	19	F	Master	4	Norway	-	-	2
	20	F	Bachelor	2	Norway	-	-	2
	21	F	Bachelor	2	Chechnya	-	-	3

^**a**^ Three nurses were supposed to attend the interview, but two had Covid-19 and could not attend

### Data collection

Data collection took place between March and December 2021. The interviews were scheduled at a convenient time for the nurses and took place in a meeting room at the University of Agder or the hospital. The interviews were conducted in Norwegian by one or two of the authors (TA and BJ). BJ is a Norwegian researcher who has contact with immigrants and published many articles related to cross-cultural care. TA is a Ph.D. candidate of Arabic background who has been living in Norway for the last 6 years. The interviews lasted between 60 and 100 min. All interviews were audiotaped, transcribed into Norwegian, and subsequently translated into English. To stimulate discussion among the participants, the interviews started with an open question: “How is your experience in caring for patients with an ethnic minority background?” The interview guide was divided into a general background section followed by thematic areas (Table 2) and a summary section. Interview data were continuously collected until no new information was forthcoming.

**Table 2:** Sample questions from the interview guide

***Theme: language and communication***
*How do you experience communication and collaboration with ethnic minority patients?* *What experiences do you have with the use of an interpreter? (Type of interpreter)* *What do you do if it is difficult to communicate with patients?* *Do you ask for help from someone who has more experience with ethnic minority patients?* *In what way is there a difference between caring for an ethnic Norwegian and an ethnic minority patient?* *What is your impression about patients’ interest and understanding of the information they receive?*
***Theme: cultural aspects***
*How do you experience those cultural differences affect the interaction with the patients?* *What are your experiences with family visits and how does it affect your job?* *People from different cultures express pain in different ways, how do you experience it?* *How do you experience challenges in relation to food and meals?*

### Analysis

Data were analyzed using a general approach of thematic analysis of transcribed narratives of the participants [46, 47]. This classical approach to data analysis is based on a sequential process of six phases that starts with the familiarization of the researchers with their data and is followed by generating initial codes of data. Phase three began when all data were initially coded and sorted into potential themes. In phase four the themes were reviewed to cohere together meaningfully and a clear and identifiable distinction between themes were achieved. Congruence with the extracted quotations and narratives were secured. This phase was followed by defining and naming the themes (phase five) and incorporating them into the report (phase six) [46, 47]. Two of the researchers read and re-read interview statements before creating initial line-by-line codes based on statement significance. These initial codes were grouped to form general themes, which were then checked against the participants’ original transcripts and reviewed for any overlaps or contradictions before the final theme labels were created. After the initial coding, the data were explored and discussed by the research team. Three main themes with two to four subthemes were discovered and led to the findings presented below.

### Rigor

Denzin’s (2008) criteria for trustworthiness, credibility, confirmability, dependability, and transferability guided the methodological judgment [48]. Credibility was strengthened by asking open-ended questions throughout the interviews, confirming that the researchers understood the participants’ replies accurately. Credibility was further strengthened by presenting summaries of previous meetings to focus group members and by amending formulations. Confirmability was attained through two of the authors conducting the data analysis, followed by peer review by another author. In addition, data were discussed by the research group. To establish dependability, an open discussion between two of the researchers was created where they read the interviews and together discussed the interpretation of the data so that their assessments of content similarities and differences were consistent over time. Regarding transferability, purposive sampling was employed, and nurses were recruited from different hospital wards. To ensure the data were reliable and transferable, the study setting, participants, findings, and conclusions were carefully described.

## Results

**Fig. 1:**
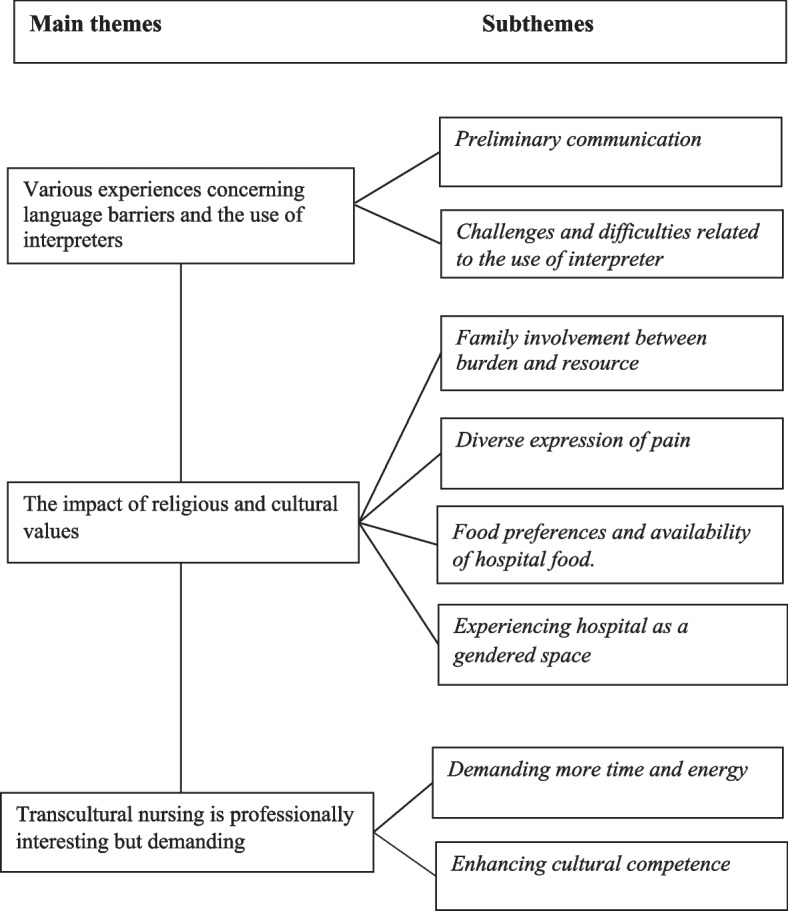
Main themes and subthemes

This study aimed to explore the nurses’ experiences in caring for patients with an ethnic minority background. The results showed that caring for patients with an ethnic minority background was both challenging and enriching. The nurses used their own understanding and preconception of care in their encounters with patients with an ethnic minority background. The following themes and subthemes emerged from the data analysis (Fig. 1).

### Various experiences concerning language barriers and the use of interpreters

This theme describes the difficulties participants encountered when communicating with minority patients and is centered around language barriers and interpreters. Language ability was a huge factor in patient care for establishing the health concern, prescribing treatment, and defining recovery. Due to the language barrier, a lot more time and effort had to be spent communicating with the patients. Background information was essential for patient care, and this was hard for participants to gather because of the language barrier.

#### Preliminary communication

The nurses encountered difficulties when communicating with patients with an ethnic minority background. Miscommunications could be very distressing for the nurses and patients alike. Nurses recounted that the first point of patient interaction with the Norwegian healthcare system is through communication with the nursing staff, either directly or indirectly through the mediation of a professional interpreter or simply a relative. Most of the nurses believed that it is an important aspect of care delivery to communicate correctly. However, their narratives of everyday interaction with the patients highlighted some challenges.

In any case, it seemed that extra energy, time, and effort were required to understand the patients, as one of the nurses recounted:

*“Some have good Norwegian language ability and can understand what we say well, while others cannot speak the language, so lots of time and energy is spent trying to read and understand them effectively.” (Participant 3)*

Through their communication, the nurses expressed their desire to do the best for the patient, as one nurse said:

*“I often get in contact with patients from a minority background. I want them to understand that we want the best for them and that we are interested in understanding their experiences with the Norwegian health system.” (Participant 5)*

After identifying a linguistic problem, nurses tried to create an interpretive system, where they tried to discover if the patient or their relatives could speak any of the familiar languages such as English or French. If so, then simple conversations such as food choices or daily care could be informally conducted, but if medical issues were being discussed it was deemed better to call an interpreter. Participants and other hospital staff with the knowledge of additional languages could also be used as interpreters. However, this process could take up their time leading to poorer patient care. Some participants relished the opportunity to assist, while others felt that this is not their job and that an official interpreter would be more appropriate. As one participant described:

*“I would have been terribly bored if I had been asked all the time to be an interpreter. That’s not why I work at the hospital.” (Participant 5)*

Due to poorer understanding of health vocabulary, either the participant or the interpreter would need to use simpler words and concepts to convey their meaning rather than technical terms. Where necessary participants have used other methods of communication such as gesturing and pointing to body parts, drawings, pictures, or videos. These were helpful stopgaps until an interpreter arrived or to illustrate the translation. When patients were left slightly uncertain about a subject, they would often be able to supplement this with internet searching on their phones. This was less likely the older the patient was, and so elderly patients were particularly vulnerable to miscommunications. Participants found control questions were important to determine if the patient actively understood what was being said to them, and participants who were themselves a minority were more persistent and likely to persevere with communication until they were satisfied that the patient understood them.

Participants reported feeling helpless at times as they lacked the means to properly converse with patients with an ethnic minority background, even when an interpreter was present. This was particularly the case where they needed to inform the patient of a complex home care condition such as diabetes.

Participants noted that patients could become distressed when communication failed, and they were let unaware of their situation.

*“I remember very well that we had a case where a patient did not know why he was receiving treatment. And it took many days before he realized why he was here.” (Participant 2)*

Further, the participants noted that minority patients from certain cultures could be very polite and kind to the point that they would downplay their symptoms or pretend to understand what is said to them.

#### Challenges and difficulties related to the use of interpreter

While nurses believed that they could handle everyday matters of food and care without much dependence on a professional interpreter, this is not the case for certain patients or diseases that are perceived as serious or otherwise dependent on correct communication. A typical situation depicted by the nurses was that of children with special needs (e.g., diabetics). Most of the nurses said that the interpreter is called upon for the visit from the doctor, and patients are advised to prepare questions in written form. They used both telephone interpreters and interpreters who come to the hospital. Later, they said that there is a large difference in the technical competence of interpreters. The importance of competence of the interpreter is linked to the perceived seriousness and vulnerability of the patients. One of the participants stated this point:

*“I think it is important to ensure the quality of interpretation and this saves time and effort as well, especially for those who work with sick newborns.” (Speaker 11)*

Participants experienced that interpreters have varying levels of competence, they underlined that the hospital should tend to use interpreters that they have had good experiences with.

*“If there is a need to use interpreter more than once, then it is better to use the same interpreter when needed. It works much better if there is a good interpreter and you use the same one to interpret to the same patient all time.” (Participant 11)*

The nurses had also experienced that some interpreters would also only convey a simplistic message rather than the full picture and would not accurately explain to the patient their situation. They found that the higher quality interpreters would make sure to convey exactly what is said by both parties without their own perceptions. Patients would occasionally pretend to understand, and the participants needed to be careful to make sure that the patient actually understood the message.

*“I have been in similar situations where patients say yes to everything without understanding any of what is being said. So often I ask them to tell me, what have I said. Or what have you understood? I ask them control questions and say something funny like, are you ugly? and they respond yes, yes. Then I know they haven’t understood anything. Then sometimes the communication becomes both funny and very difficult at the same time.” (Participant 3)*

On occasions participants found that the interpreters would refuse to tell a patient that they have cancer or are dying. This necessitated the use of other interpreters. Interpreters tended to be more phone based due to Covid-19, participants felt this was both safer and more convenient for all involved. This shortened the time between patient admission and an interpreter beginning translation. However, this created other issues as the interpreters were unable to view body language (unless a webcam was provided) and would miss nuances that would be noticeable. There could also be the issues of internet or signal disruption.

Participants and other hospital staff with additional languages could be used as interpreters however this could take up their time leading to poorer patient care. Some participants were happy to act as an interpreter and would relish the opportunity to assist, while others felt that this was not their job and that an official interpreter would be more appropriate.

*“I would have been terribly bored if I had been asked all the time to be an interpreter. That not why I work at the hospital, I work there to be a nurse, not to be an interpreter. I understand it if there is an emergency, and then they can be used, but otherwise we have phones and interpreters, and I we should use them.” (Participant 5)*

Another participant suggested:

*“I suggest that in the hospital data bank we should have a list with the staff and the languages they speak, so that one can somehow contact them and use them as an interpreter in emergency cases or when an interpreter cannot be reached.” (Participant 9)*

Nurses also tried to explain why their patients may or may not be willing to opt for the interpreter. The reason might be that the patient is not willing to incur delays in the provision of care and treatment due to the interpreter’s “gatekeeper” role. Similarly, the nurses mentioned that sometimes the interpreter is from a small community of people and knows the patient personally. Patients may opt to reject an interpreter because they want confidential treatment and to not expose their medical problems to the rest of their community. One of them told:

“*Even though the interpreters have a duty of confidentiality, but it could be that patients don’t trust them, and they are afraid that others in their community will know about their situation.” (Participant 10)*

Similarly, some patients might have a value framework that is based on the lines of patriarchy. For example, a father did not want to use an interpreter and he would interpret to protect his wife and children from any bad news they might receive. In addition, some female patients might not use a male interpreter for their gynecological issues.

*“And then you experience those relatives who in a way do not want an interpreter; I have experienced that. The father can do so much, he thinks that is good enough. He understands, he conveys, and then I may find that I do not get the information I really want. But still, in a way, they do not want an interpreter. And what do you do then? Maybe it’s because he’s keeping that information and protecting his wife from the truth, and he thinks it’s better for her to not know about her baby’s situation.” (Participant 12)*

### The impact of religious and cultural values

This core theme demonstrates the cross-cultural encounters of nurses with patients’ need for diverse foods, visits by relatives, expression of pain, and gender-related sensitivity. Some of these diversities are connected both to religious beliefs and cultural traditions. Participants found that religion was often something that they needed to cater towards. Cultural and religious rituals were disruptive to participants as they could make the act of eating, giving birth and other processes longer and more complex, putting additional demands on the participants’ time. When dealing with end-of-life care and funeral arrangements some minorities had in-depth cultural or religious arrangements. Participants have had to accommodate this where possible; they would ask patients in advance if there was anything they needed. Conversely some participants found that patients from certain minorities would not show grief or discuss the bereavement at all as this was their cultural norm. Cultural perceptions would come into conflict with the participants when patients would disagree with the advice or treatment as they have different opinions.

#### Family involvement between burden and resource

Some participants found that minorities requested that their family be informed about their health situation as that is their cultural preference. The patients would often have large families who would visit, and they were included in the patient’s confidentiality. However, this caused problems when the patient was stressed, tired or otherwise not at their best as that many people would be overwhelming. Patients would struggle to rest and would decline as a result. Participants needed to restrict the number of visitors for this reason. However, they experienced that families had a larger role in caring for patients with an ethnic minority background.

There exists a consensus among the nurses that patients of foreign origin tended to have a greater number of private visits by family and friends. These situations create challenges for the nursing staff, yet others believed that it also offers opportunities to facilitate the patient mainly in terms of cultural adaptation to the Norwegian system. Challenges of family visits are mostly related to the privacy of other patients and the duration of the visits. The situation creates a kind of alienation for the nursing staff because, on the one hand, the visitors are able to provide physical moral and linguistic support, yet on the other they may create an additional burden for the system. As one participant described:

*“There have been challenges related to that, also because the hospital mostly has double rooms. We have few single rooms. Neighboring patients are often affected by the fact that there are so many visitors, and they are there for a long time.” (Participant 3)*

During the period of the Covid-19 pandemic, these visits were perceived as a challenge and a threat; hence, the visit timings were limited to half an hour, with strict procedures. After a long period of lockdown, duration and frequency of visits were increased again. The nurses had the impression that family presence is deeply connected to gender and culture, especially when it comes to the hospitalization of the newly migrated, less integrated females.

“*The husband wants to be there with his wife. Sometimes it is not easy for the man to be there, and it is not allowed, so the man gets a chair to be able to be there all night; that’s weird to me.” (Participant 4)*

As explained by the same participant, this might be because the patient is afraid or needs physical, emotional, or linguistic support from her husband who is more accustomed to the cultural norms of hospitalization in Norway.

Similarly, another advantage mentioned by the nurses consists of food that is offered by the relatives to the patients and is readily accepted. They experience that patients can have a multitude of food desires and conceptions of which food is both good and bad.

Almost all the participants were well aware of the problematic outcomes attached to the family visits. They believed the biggest issue was the number of visitors. Sometimes they were not prepared to handle 5 or 6 visitors, and thought it to have a negative impact on the patient. Narrating the case of a mother who gave birth to an underweight baby one of the participants said:

*“The poor mother was quite tired (of visitors), and as soon as one had left, the next one would come, and we were not prepared to stop this. That was not good for that baby or for the mother, or for the other babies there on the ward.” (Participant 11)*

Many of the participants reported that the minority patients’ families were very insistent on bringing their own food. This was due to a number of reasons including: dietary requirements, religious grounds, or cultural reasons. The most common reason tended to be that in the patients’ country of origin hospitals would not provide food and the patients’ families would need to do this. Participants needed to reassure the families that the hospital would provide food and were equipped to handle dietary requirements and religious diets.

*“I remember when we were in Malaga in Madagascar 3 years ago, it was mandatory for relatives to take care of the patients, otherwise they were not cared for, and I think there is a part rooted in people that they must join in. In Madagascar, they are also responsible for cooking. There were lots of people out in the courtyard cooking around for the patients. And if they did not, then the patient did not get food.” (Participant 9)*

#### Diverse expressions of pain

Participants claimed that minority patients were known to exaggerate the amount of pain they seemed to be experiencing. As one participant mentioned:

*“But I think it's something cultural, that they do it to get treatment, to be prioritized. And they think the system is like that. They think that we have a system where those who cry the loudest get help first. That is the impression one gets.” (Participant 9)*

According to the participants, patients from some cultures were more expressive than others when it comes to pain. Some may amplify and verbalize while others may attenuate the expression of pain and be silent.

*“One patient may be sitting in bed listening to music while he’s in pain, and another shouting and screaming.” (Participant 1)*

Sometimes the expression of pain became violently intense, as might be the case in the presence of close relatives and visitors. Some nurses admitted that they had difficulty dealing with expressions of pain in many cases. Especially when it comes to some Asian cultures, people tended to hide their emotions, and pain is one of them. As one participant told:

*“What is challenging is that some are very quiet; it is not easy to understand those signals, that they are seriously ill when they arrive at the hospital. So, you try to ask them where on the pain scale they are, and you have to describe the pain.” (Participant 3)*

Some participants believed that the trauma of pain can be mitigated by overcoming the barriers of language. Language barriers and the expression of pain go hand in hand. As one described:

*“By explaining what the patient is about to go through, you create a less or non-traumatizing experience.” (Participant 14)*

Participants found that the exaggeration or restraint in pain expression often stemmed from fear due to the unfamiliar country and language. Overall, the different ways that patients from an ethnic minority background expressed pain made it difficult for participants to assess the patients.

Pain expression can also extend to psychological conditions, one participant noted that:

*“In my experience, people that experienced war tend to have more psychological issues as well as physical pain. By comparing Norwegian patients and foreign patients, I have experienced that, Norwegian patients are less violent when experiencing a migraine for example, as they only tend to lay their hand on their forehead when experiencing pain. Foreign patients, however, tend to be more violent, hit their pillows and scream louder. Usually, patients that experienced war, express pain more intensely.” (Participant 16)*

#### Food preferences and availability of hospital food

Food preferences were frequently noted by participants for example, Muslim patients were not able to eat pork. This was problematic when the hospital did not have many food options. In this situation most patients with an ethnic minority background would bring food from home, and that could cause some discomfort to other patients if the food is particularly strong smelling and spicy.

Given the fact that food is important, some nurses tried to settle the question of food in their opening interaction with the interpreter so that there was no room for confusion. The nurses had a clear understanding that patients with an ethnic minority background may have diverse needs for food at the hospital. Some patients brought their own food to the hospital as might be the norm in their home country. They were not aware that food would be provided for them by the hospital. However, patients’ food-related needs could only be partially fulfilled by the hospital kitchen, and kitchen staff also experienced challenges:

*“Meals are always a challenge. Some patients solve this by having their relatives bring food for them, which I think is fantastic.” (Participant 18)*

The nurses mentioned that linguistic problems and failure to express their choices might make them skeptical, especially when it has to do with taboo foods in the migrant patients’ culture of origin. One participant said:

*“We ask the patients what they would like to eat. Many Muslims say they eat everything but not pork; we make a note of it, and then we order something without pork.” (Participant 5)*

Some participants have shown their concern for food that is not produced in the hospital kitchen. First, this food may be brought to the hospital facility by the relatives of the patients who may force the patient to eat the food when it is not desired. Second, the food may cross-contaminate or be very aromatic, which creates a sense of deprivation for other patients admitted to the hospital.

#### Experiencing hospital as a gendered space

Participants found that patients would often prefer a same sex staff member for their care. They experienced taboo regarding undressing which was sometimes problematic when it came to participants examining patients or for breast feeding. Participants often needed to take extra steps to cater to patients with an ethnic minority background patient’s modesty.

There was a frank understanding that many female patients of foreign origin will reject the nursing support of male nurses. Providing personal care may include some sort of physical handling and touching, which is conceived as an asexual act within Norwegian culture but not in the case of ethnically diverse patients. Sometimes a male patient will not want a female nurse either. This links to their comfort in having the presence of their relatives in the hospital.

*“It actually goes both ways; some women do not want a male nurse for example, but it can also go the opposite way where male patients do not want a female nurse because they do not trust them.” (Participant 7)*

To the extent of interpreting language related to sexuality and gynecology, some patients did not accept a male interpreter. Some nurses experienced norms of patriarchy. As one participant described:

“*In some cultures, the father is responsible for his family; he is the one who decides everything. We noticed that he wants to interpret for his family and the mother doesn’t want to have an interpreter.” (Participant 11)*

### Transcultural nursing is professionally interesting but demanding

This core theme is a result of everyday encounters and a long-term process of acquiring cultural competence by the nurses. Caring for patients with an ethnic minority background might be demanding, but the nurses also expressed it as an enriching and worthwhile experience. One of the participants said: “*I often actually choose foreign patients.” (Participant 11).*

#### Demanding time and energy

Sometimes it was a challenging and demanding job to go the extra mile in catering to linguistic and cultural needs. As one of the participants said:

*“Minority patients may not understand that we have a limited amount of time to communicate with them. As a result, this may lead the patients to feel unsafe and believe that they are not being taken care of.” (Participant 15)*

Another participant noted how taking care of patients with an ethnic minority background requires both time and effort:

*“I sometimes feel that I have to take a break, because it is quite demanding, and it takes a lot of time and in a way, you don’t always get the time in the department to do it, then I say no, now I cannot. Now I have to take a break.” (Participant 12)*

#### Enhancing cultural competence

Most participants believed that having relatives in the hospital was a cautiously advantageous situation because relatives will help the patient comply, feel safe, and accept physical support saving the precious time of the staff of the hospital. Many participants believed that they had the competence and skills to handle cultural diversity in the hospital and that it was exciting to experience this diversity. One participant expressed that she has had training that was specifically about cultural differences, and she said:

*“I remember that we had lessons about why patients from different cultures acted the way they did, and why some brought their own food and family with them. I found it very interesting.” (Participant 16)*

The majority of nurses said that they had come to terms with their foreign patients through personal experiences and diversity-based encounters, which added up their cultural competence.

According to the respondents, dealing with cultural diversity provided them with an opportunity to cater to the diversity of care and this process would enhance their ability to understand the values, beliefs, norms, linguistic problems, and expressions of cultural life.

Nurses with an ethnic minority background, nurses who have experienced working in diverse cultures, or those who were in an exchange student program, tend to find it easy to deal with immigrant patients. They found it easy to blend their own culture to fit within the culture of the patients with an ethnic minority background.

## Discussion

The present study was conducted with the aim of exploring nurses’ experiences in caring for patients with an ethnic minority background in a hospital setting. The challenges arose from difficulties surrounding communication which could be further complicated when they were paired with issues of religious and cultural values. The data imply that nurses have a good sense of everyday communicational and cultural challenges in caring for patients with an ethnic minority background. These challenges have also been described by the nurses as opportunities to interact, grow and enrich their own cultural competence.

As to the communicational experiences of the nurses, it can be inferred that everyday interactions are not disturbed by the mere linguistic gap, and the need for the interpreter is required by nurses, depending on to the nature of the disease (e.g., diabetes) or the patient (e.g., newborn). These findings are supported by earlier studies that revealed nurses use mobile apps and gestures to communicate with patients on a daily basis [49, 50]. It can be inferred from the findings that opting for an interpreter is not a ritualistic and ubiquitous stage of the caring process. In fact, this decision is taken by the patients on many pragmatic grounds including the perception of delays, miscommunication, omission, and breach of privacy. These findings are concomitant with studies conducted in the past [23, 25, 50]. The findings also confirm that the communicational and linguistic gap was perceived by the nurses as a major threat to the timely provision of health care [23, 51]. Consistent with previous research, the findings of this study suggest that using interpreters is not free from limitations, since interpreters may not always be familiar with medical terminology and may find it difficult to explain certain concepts to patients. In addition, some interpreters could provide the patient with an oversimplified message rather than the whole picture, meaning they fail to fully explain the situation [23, 52]. In the absence of clear and direct communication, recognizing how patients with an ethnic minority background express their pain was an important cognitive technique for the nursing staff [53–55]. Effective communication has the potential to alleviate pain through a sense of comfort. These findings are consistent with earlier work that suggested that patients from Asian cultures tended not to verbalize pain, whereas those from other cultures tended to have more pronounced and vocal expressions of pain [56, 57].

One dimension of interaction characterized by cross-cultural experiences is that of visits by family and friends. The findings of the study imply that cultural diversity at the hospital offers its own learning opportunities for nurses regarding family visits. For instance, family visits were seen as a threat to the privacy of other patients, a source of contamination, and a waste of time. Yet, they can also be seen as a source of resources, physical, and moral support for the patient, essentially complementing the role of nurses. This finding is consistent with earlier work on hospital visitations that showed that nurses have general acceptance of visitations in general wards but not in intensive care units [32]. Family visits are also associated with the acceptability of home-prepared food brought to the hospital by visitors [58]. The nurses described that this could have been the case for newly migrated families who were not aware that food is provided to patients by the hospital. Nonetheless, patients’ perception of food might be linked with underlying permissible ingredients in their religion. Hospitals operate under certain food standards, and it is not possible to meet all desires when it comes to food. Food diversity is linked to cultural sensitivity, which leads to self-perceived cultural competence among nurses [59].

In the case of our results, and in some previous studies, there exists gender-related attitudes, biases, and behaviors that are revealed when hospitalization is required [60–62]. These assumptions can only partially be verified in the Norwegian context through the present research. However, it is pertinent to show that the presence of an intimate partner for the physical support of female patients and the rejection of male interpreters in gynecological problems testifies to the presence of gender biases among patients [63, 64]. The same holds true for the refusal of support from a nurse of the opposite sex [65, 66]. These gender related issues can be connected to the patient’s religious faith and cultural traditions. Many females are also reluctant to talk about gynecological problems because they believe its equal to the expression of nudity or because in their countries of origin, gynecologists are predominantly female [65].

Our results show that encounters with cultural diversity create some obvious challenges in time management, but it can also offer opportunities for self-reflection and enrichment [67]. These encounters create a kind of diversity-related sensitivity and teach skills for introspection and values such as patience, and humbleness [68]. Successful encounters with culturally diverse patients link the understanding of values, norms, and beliefs of the patient with communicational expression and verbalization of pain and gender-related issues. These findings are supported by studies conducted across various European countries [57, 69].

The participant nurses who were from a cultural or ethnically diverse background other than Norwegian, had worked in another country, or had been in a student exchange program tended to display higher levels of cross-cultural sensitivity and were more likely to express positive outcomes and cultural sensitivity than the other participants. These findings are similar to those of several other studies and illustrate the importance of implementing exchange programs in nursing education [5, 70–72].

### Strength and limitations

To our knowledge, this is the first Norwegian study to reveal that caring for patients with an ethnic minority background is both demanding and enriching. The interviews were conducted by two researchers, one Norwegian and the other with an Arabic cultural background. The participants all worked in Norwegian hospitals, some had a different cultural background, and some had worked or lived in another country. This diversity enriched the discussion during the interviews.

Due to Covid-19 two out of the three participants in one of the groups were unable to attend the scheduled meeting. The interview was therefore conducted as an individual interview. This could be considered as a limitation. Data were transcribed into Norwegian and translated into English because subsequent analysis and report writing were planned to be published in English. Translation was carried out by author TA whose ethnicity is not Norwegian, hence all translations were proofread by author BJ to ensure the accuracy of the translations.

## Conclusion and recommendations

The purpose of this study was to explore nurses’ experiences in caring for ethnic minority patients. Throughout, the participants expressed that caring for ethnic minority patients offers both challenges and opportunities to enhance cultural competence and cultural sensitivity among nurses. Our findings highlighted three main themes: various experiences concerning language barriers and the use of interpreters, the impact of religious and cultural values, and caring for patients with an ethnic minority background is professionally interesting but demanding.

First, participants described difficulties when communicating with ethnic minority patients, which centered on language barriers and the use of interpreters. Language ability was a huge factor in patient care. The language barrier made interacting with patients more time- and energy-consuming. Second, the cultural demands relating to specific foods, visitations, gender, and expression of pain are interlinked and offer a substantive opportunity for organizational learning and enrichment. The encounters with diversity, challenge both patients and nursing staff alike. Dealing with patients in a cross-cultural situation requires investment in both skills and time. Finally, despite the fact that caring for ethnic minority patients might be demanding, nurses described it as an enriching and worthwhile experience. Nursing ethnic minority patients increases the learning skills of the nurses.

This study showed the importance of educational programs directed toward cultural competence in nursing education. As emphasized by nurses, information on the Norwegian healthcare system and how it works should be supplied to new migrants. Further studies of cultural competence among healthcare providers are warranted to explore their cultural sensitivity and improve cross-cultural awareness.

## Data Availability

The datasets used and/or analyzed during the current study are not publicly available due to General Data Protection Regulation laws but are available from the corresponding author on reasonable request and with permission from the Norwegian Centre for Research Data.
